# Systematic evaluation and meta-analysis of quality and safety of life in patients with chronic rhinosinusitis treated by acupuncture: A protocol for a systematic review

**DOI:** 10.1097/MD.0000000000018965

**Published:** 2020-02-28

**Authors:** Lu Li, Shouliang Hu, Xin Zhu, Tianmin Zhu, Hui Li

**Affiliations:** a Chengdu University of Traditional Chinese Medicine; b Chengdu University, Chengdu, China.

**Keywords:** acupuncture, chronic sinusitis, meta-analysis, systematic

## Abstract

**Background:**

This study aims to assess the quality and safety of life in patients with chronic rhinosinusitis (CRS) treated by acupuncture.

**Methods:**

An extensive database search was executed to find the randomized controlled trials on acupuncture vs. sham acupuncture, and any other treatments for patients with CRS. Review Manager 5 (RevMan) was used for the data analysis. A strict methodology was used when the meta-analysis was performed.

**Results:**

This study systematically assesses the effectiveness and safety of acupuncture in patients with CRS. The primary outcomes include health-related quality of life, disease severity, treatment-related adverse events. The secondary outcomes are comprised of recurrence rate, endoscopic score, computerized tomography (CT) scan score and objective physiological measures.

**Conclusion:**

This article summarizes the current evidence base for the quality of life and safety of acupuncture in patients with CRS.

**Systematic review registration:**

International Prospective Register of Systematic Reviews PROSPERO (registration number CRD42018114432).

## 1 Indroduction

Chronic rhinosinusitis (CRS) is one of the most common inflammatory disease of superior respiratory tract, with a reported prevalence of 10.9% in European^[1]^ and 11.8% to 17.4% in American.^[2]^ It is defined by EPOS 2012^[3]^ as symptoms that last more than 12 weeks and present with 2 or more symptom, including nasal blockage/obstruction/congestion/or nasal discharge, facial pain or compression, reduction or loss of smell. CRS have been subclassified into 2 groups: CRS with nasal polyps (CRSwNP) and CRS without nasal polyps (CRSsNP). Aside from getting wind for its negative impact on an individual's quality of life, CRS is connected with patient's medical and surgical resource consumption dealing to a lot of medicine spending. According to the research report, the annual medical expenditure of the us medical system for CRS exceeds 22 billion dollars, which brings a huge economic burden to the us government.^[4]^ Currently, treatments for CRS include drug treatment (mainly antibiotic, antihistamines, or corticosteroids), and sinus surgery,^[3]^ but the evidence is limited and the results disappointing.^[5]^ Moreover, patients who reuse antibiotics and sinus surgery have an increasing risk of drug resistance and surgical complications.^[6]^ Perhaps due to the limited of these therapy and the feature of the condition, alternative medicine is becoming increasing common and frequently used by patients with CRS.

Acupuncture, defined as an intervention of stimulates acupuncture point on the body with needles,^[7]^ was 1st documented in Huangdi's Orthodox Classic, which trace back to 100 BC. Traditional Chinese medicine believes that meridian is the channel through which qi and blood run in the human body. Acupuncture can make the blocked meridian unblocked and make it exert normal physiologic functions, regulate the balance of Yin and Yang of the body, assist the body to regulate qi and dispel diseases and evils. It is a safe treatment and is often used to relieve sinusitis and nasal symptoms.^[8]^ Acupuncture is one of the most extensively used complementary therapies for a variety of chronic conditions in plenty of countries.^[9]^

How acupuncture does relieve the symptom of sinusitis and nasal for CRS is still unclear; however, emerging article shows acupuncture might work through several possible mechanisms. The article shows that antiinflammatory effect of acupuncture are mediated through the reflexive central inhibition of the innate immune system.^[10]^ Modern studies^[11]^ have shown that acupuncture can improve the body immunity ability of mice with CRS and repair the nasal mucosa injury caused by inflammatory response. In mice CRS model, acupuncture reduces the protein expression of NF-κBp50, inhibits the production of many inflammatory mediators, leading to the relief of inflammatory damage in nasal sinus mucosa, correct Th1/Th2 imbalance, and promote inflammation self-healing. Another theory is about the release of analgesic states in the central nervous system, which is that acupuncture regulates the production of endorphins, serotonin, and acetylcholine.^[12]^

There is an increasing use of acupuncture in the management of CRS with inconclusive results. The main attraction of acupuncture for patients with CRS and health professionals is that it is generally cheap and relatively painless, with no adverse effects if treated by qualified medical staff. Although public interest in the use of acupuncture to enhance effectiveness has arisen from time to time, evidence of its effectiveness is still needed. A systematic review of the use of acupuncture in CRS to determine its effectiveness and safety will address these needs.

## 2 Objectives

The objective of this review will be to assess the HRQOLL and safety of acupuncture compared with placebo, no intervention, or other interventions in CRS.

## 3 Methods

### 3.1 Types of studies

All randomized controlled trials (RCTs) comparing acupuncture with placebos, no intervention, or any other interventions in patients with CRS will be included. Trials published as abstracts only will be excluded. We are limited to include only those published in English and Chinese.

### 3.2 Types of participants

We will include patients with CRS with nasal polyps (CRSwNP) or CRS without nasal polyps (CRSsNP) diagnosed by the European Position Paper on Rhinosinusitis and Nasal Polyps.^[3]^

We will exclude studies that have included a majority of patients with: cystic fibrosis, allergic fungal sinusitis/eosinophilic fungal/mucinous rhinosinusitis, antrochoanal polyps (benign polyps originating from the mucosa of the maxillary sinus), malignant polyps, inverted papilloma, and primary ciliary dyskinesia.

### 3.3 Types of interventions

We will include manual acupuncture, electroacupuncture, and fire or warm needling. The types of stimulation can be the hand, electricity, and moxibustion. Control interventions including: no intervention, sham acupuncture, drug therapy, operative treatment, and other treatment.

### 3.4 Types of outcome measures

#### 3.4.1 Primary outcomes

1. Health-related quality of life, using disease-specific health-related quality of life scores, including the Sino-Nasal OutcomeTest-22 (SNOT-22), Rhinosinusitis Outcome Measures-31 (RSOM-31), and SNOT-20.
2. Disease severity, as measured by patient-reported symptom score (such as the Chronic Sinusitis Survey [CSS] questionnaire and visual analog scales). In the absence of validated symptom score data, we reported patient-reported individual symptom scores for the following symptoms: nasal obstruction/blockage congestion, nasal discharge (rhinorrhea), facial pressure/pain, loss of sense of smell (adults), and cough (children).
3. Treatment-related adverse events.

#### 3.4.2 Secondary outcomes

1. Recurrence rate.
2. Endoscopic score (depending on population, either nasal polyp size score or endoscopy score, e.g., Lund–Mackay/Lund–Kennedy).
3. Computerized tomography (CT) scan score (e.g., Lund–Mackay).
4. Objective physiological measures: nasal peak flow, nasal volume, nasal cross-sectional area, nasal nitric oxide (nNO), and ciliary function (including saccharine clearance time).

### 3.5 Search strategy

The systematic search will be conducted with English and Chinese. We will search the Cochrane Central Register of Controlled Trials (CENTRAL) in the Cochrane Library, MEDLINE Ovid, Embase Ovid, and Science Citation Index Expanded (Web of Science). We will also search 3 Chinese databases: the China National Knowledge Infrastructure (CNKI), Chongqing VIP (CQVIP), Wanfang Date. All databases will be searched from the build database to October 25, 2018 using a predetermined search strategy. The proposed retrieval strategy is given in the appendix.

### 3.6 Data collection and analysis

#### 3.6.1 Selection of studies

Two review authors (LL and SLH) will use uniform standards to independently screen titles and abstracts retrieved from databases to select inclusion of potentially eligible trials. If it appears relevant to the review theme, they will obtain and evaluate the full text. When disagreements arise, we will discuss them on the basis of inclusion and exclusion criteria or adjudicate by 3rd author (XZ).

#### 3.6.2 Data extraction and management

The two review authors (LL and SLH) will independently extract data from the included experiments via a standardized electronic data collection, and the differences will be resolved by consensus or discussion with third author (XZ). If the needed data is not mentioned in the article or there is any uncertainty, we will try to contact the authors of the trial.

We will extract the following data from each included trial: study characteristics (authors, year of publication, journal), trial design (including random method, allocation concealment, blind method design), patient characteristics (the total sample size, average age, gender, race, course of disease, eligibility criteria), intervention group and control group (therapies, frequency of the therapies, treatment duration, number per group, follow-up time), type of adverse effects, treatment setting (e.g., community, general hospital, specialized hospital).

#### 3.6.3 Dealing with missing data

When data were missing, we planned to solve it in the following ways. We will contact study authors to obtain the missing data via phone or e-mail. If this was impossible, we will analyze data on an intention to treat (ITT) principle basis, as it is more likely to reflect truth if patients do not participate in follow-up reviews.^[13]^ We can approximate standard deviation using the standard estimation methods from standard errors, exact *P* values, or 95% confidence intervals (CIs).^[14]^ We will perform a sensitivity analyze, and the potential influence of missing data will be addressed in the finding of the review.

### 3.7 Assessment of risk of bias in included studies

The Cochrane “Risk of bias” assessment tool^[15]^ will be used to assess the included randomized controlled trials for risk of bias. Methods of individual trials will be assessed for bias risk in the following areas: selection bias (e.g., sequence generation and allocation concealment), performance bias (e.g., blinding of participants and personnel, other potential threats to validity), detection bias (e.g., blinding of outcome assessment, other potential threats to validity), attrition bias (e.g., incomplete outcome data), reporting bias (e.g., selective outcome reporting). And all included trials will be divided into the following categories: “low,” “high,” “unclear” risk of bias. Two review authors will independently assess these domains, and any disagreements will be resolved by discussion or by a 3rd author (XZ).

### 3.8 Measures of treatment effects

We will use Review Manager 5 (RevMan) for data analysis.^[14]^ For continuous data, mean difference (MD) will be used to calculate the overall effect size, if different methods and scales calculate the same outcomes variables, the standardized MD (SMD) with 95% CIs will be used to measure the treatment effect. For categorical outcomes, the risk ratio will be used to assess the treatment effect with 95% CI.

### 3.9 Data synthesis

Review Manager 5.3^[14]^ will be used to conducted all meta-analyses. We will use the Chi-squared test and *I*^2^ statistics to analyze statistical heterogeneity of different studies.^[16]^ And according to the recommendation of Cochrane systematic evaluation,^[15]^ if the *P*-value of the Chi-squared test for heterogeneity was >0.10 or *I*^2^ < 50% would consider its statistical heterogeneity acceptable. If studies included are homogeneous in the field of extracted information, we will use fixed-effect model. If the *P*-value of the Chi-squared test for heterogeneity was ≤0.10 or *I*^2^ ≥ 50%, we think that statistical heterogeneity is high. To find the causes of heterogeneity included in the study, a sensitivity and subgroup analysis and meta-regression compare studies from different information, including age, course of disease, acupuncture treatment, and the classification of CRS. The descriptive analysis will be used rather than meta-analysis. There is high level of statistical heterogeneity of unknown cause, and the random-effects model can be used to estimate the average amount of effect for multiple studies with different or variable truth values.

### 3.10 Ethics and dissemination

This study does not need ethical approval because there is no personal data involved. The study will be published in a peer-reviewed journal.

## 4 Discussion

This systematic review will provide an assessment of acupuncture therapy in patients with CRS. Currently, drugs (antibiotic, antihistamines, or corticosteroids) and sinus surgery remain the finest-line ways for CRS treatment. Whether acupuncture can be useful as a substitution therapy for CRS is unclear. At present, no systematic review on acupuncture for CRS has put into effect a meta-analysis to compare HRQOLL and safety across various therapies. The results will explore the efficacy and safety of an economic therapy of CRS, which has not been done in the previous review. The verdict of this study will be useful for patients with CRS, clinicians, and policy-makers. This further helps to reduce the heavy burdens of patients with CRS and its syndrome on national health budgets.

## Author contributions

**Conceptualization:** Lu Li, Shouliang Hu, Hui Li.

**Data curation:** Lu Li, Shouliang Hu, Hui Li.

**Formal analysis:** Lu Li, Shouliang Hu.

**Funding acquisition:** Hui Li, Tianmin Zhu.

**Investigation:** Lu Li, Shouliang Hu.

**Methodology:** Lu Li, Shouliang Hu, Xin Zhu.

**Project administration:** Hui Li, Tianmin Zhu.

**Resources:** Lu Li, Shouliang Hu.

**Software:** Lu Li, Shouliang Hu.

**Supervision:** Hui Li, Tianmin Zhu.

**Validation:** Lu Li, Shouliang Hu, Xin Zhu.

**Visualization:** Lu Li, Shouliang Hu, Xin Zhu.

**Writing – original draft:** Lu Li, Shouliang Hu, Xin Zhu.

**Writing – review & editing:** Hui Li, Tianmin Zhu.

Lu Li orcid: 0000-0003-4932-958X.
